# Clinical significance of symptomatic and incidental venous thromboembolism in patients with cancer treated with apixaban

**DOI:** 10.1016/j.rpth.2026.106968

**Published:** 2026-09-14

**Authors:** Parwana Hussaini, Trine-Lise Larsen, Waleed Ghanima, Anders Erik Astrup Dahm

**Affiliations:** 1 Institute of Clinical Medicine, University of Oslo, Oslo, Norway; 2 Department of Hematology, Akershus University Hospital, Lørenskog, Norway; 3 Clinic of Internal Medicine, Østfold Hospital, Grålum, Norway

**Keywords:** anticoagulants, apixaban, incidental venous thrombosis, neoplasms, symptomatic venous thrombosis

## Abstract

**Background:**

Clinical characteristics and outcomes of patients with cancer with symptomatic vs incidental venous thromboembolism (VTE) remain poorly described.

**Methods:**

In this *post hoc* analysis of the apixaban as treatment of venous thrombosis in patients with cancer (CAP) study, we explored patients with cancer with VTE who received apixaban 5 mg twice daily for 6 months and then 2.5 mg twice daily for 30 months. Patients were classified as having symptomatic or incidental VTE at inclusion. The main outcomes were recurrent VTE and clinically relevant bleeding. Baseline characteristics and outcomes were compared between the 2 groups during a 36-month follow-up.

**Results:**

Of 284 patients, 163 (57%) had incidentally detected VTE. Compared with symptomatic patients, incidental patients had lower body mass index, more metastatic disease (79% vs 57%), and more pulmonary embolism or abdominal vein thrombosis. Deep vein thrombosis of the leg was more frequent in the symptomatic group (45.4% vs 7.4%, *P* < .001). There were more colorectal cancers in the incidental group (31.9% vs 19.0%, *P =* .01) and more hematological (14.1% vs 5.5%, *P =* .01) or breast cancers (14.9% vs 5.5%, *P =* .01) in the symptomatic group. Recurrent VTE occurred in 9.8% of incidental and 6.6% of symptomatic patients (subdistribution hazard ratio, 1.5; 95% CI, 0.6-3.5). Clinically relevant bleeding was more frequent in the incidental group (24.5% vs 13.2%; subdistribution hazard ratio, 1.9; 95% CI, 1.1-3.4). In the multivariable analysis, low hemoglobin and incidental VTE were independently associated with bleeding.

**Conclusion:**

The characteristics and outcomes in patients with incidental VTE differed from those with symptomatic VTE. While VTE recurrence was similar between the two groups, incidental VTE was associated with a higher risk of bleeding.

## Essentials

• Optimal management of incidental venous thromboembolism (VTE) in patients with cancer is unclear.
• We compared cancer patients with incidental and symptomatic VTE.
• Patients with incidental VTE differed clinically but had similar recurrence risks.
• Incidental VTE was associated with a higher risk of clinically relevant bleeding.

## 1 Introduction

The risk of venous thromboembolism (VTE) is higher in patients with cancer than in the general population [[1], [2], [3], [4]]. Patients with cancer with VTE have a higher rate of mortality than patients with cancer without VTE, and a higher risk of recurrent thromboembolism than patients with VTE without cancer [5,6]. VTE is often asymptomatic and detected incidentally when imaging is performed for cancer diagnosis, staging, and follow-up. However, it is important to note that some incidental findings, particularly pulmonary embolism (PE), may represent imaging artifacts rather than true embolic events in nondedicated computed tomography (CT) scans. Routine thoracic CT scans performed with contrast timing optimized for the aorta rather than the pulmonary arteries can produce flow-related artifacts that mimic PE. A meta-analysis found that 3.4% of oncological patients had incidental PE [7]. Knowledge about the optimal management of incidental VTE in these patients is limited. A prospective observational cohort study of 695 patients with cancer with incidental PE reported a 1-year incidence of recurrent VTE of 6% despite ongoing anticoagulant therapy with low-molecular-weight heparin, reaffirming the clinical significance of such events [8]. Two recent clinical trials on anticoagulation therapy for VTE in patients with cancer investigated the differences between incidental and symptomatic VTE [9,10]. Both studies reported a substantial risk of recurrent VTE in patients with incidental VTE treated with anticoagulant therapy, supporting current guideline recommendations to treat patients with incidental and symptomatic VTE similarly [[11], [12], [13]]. According to these studies, patients with cancer with incidental VTE showed a lower incidence of recurrent VTE and a higher incidence of major bleeding than those with symptomatic VTE.

In the current study, we aimed to compare incidentally detected VTE with symptomatic VTE in patients with cancer treated with apixaban with regard to patient characteristics, risk of recurrent VTE, and bleeding.

## 2 Methods

This is a post hoc analysis of data from the apixaban as treatment of venous thrombosis in patients with cancer (CAP) study. The rationale, design, and results of the CAP study have been described previously [14,15]. Briefly, the CAP study is a prospective, single-arm, interventional study conducted in Norway to assess the efficacy and safety of apixaban as a treatment for cancer-associated VTE. The trial is registered with the identifier NCT02581176 in ClinicalTrials.gov. Written informed consent was obtained from all patients. Ethics approval was obtained from the Norwegian Regional Committees for Medical and Health Research Ethics. The study was performed in accordance with the principles of the Declaration of Helsinki and Good Clinical Practice.

Detailed inclusion and exclusion criteria have been presented previously [14]. Inclusion criteria were patients aged ≥18 years with a cancer diagnosis or treatment (excluding basal cell or squamous cell carcinoma of the skin) within 6 months before enrollment and an objectively confirmed VTE (both symptomatic and incidental, regardless of site). Exclusion criteria were previous therapeutic anticoagulation for more than 96 hours before study entry; platelet count < 50 × 10^9^/L; creatinine clearance < 30 mL/min; active bleeding, high bleeding risk, or a bleeding risk deemed greater than the risk of untreated VTE; and concurrent use of strong inhibitors or inducers of CYP3A4 or P-glycoprotein.

The treatment schedule was apixaban 10 mg twice daily for 7 days, followed by 5 mg twice daily for 6 months and 2.5 mg twice daily as long as the treating physician found it necessary. The patients were followed up for a total of 36 months. The endpoints were recurrent VTE, major bleeding, and clinically relevant nonmajor bleeding (CRNMB) after treatment with apixaban for 6 months and 36 months.

Incidental VTE was defined as events detected on imaging tests performed for reasons other than clinical suspicion of VTE as judged by the treating physician. Recurrent VTE was defined as a new symptomatic or incidental deep venous thrombosis (DVT) or PE or unexplained death for which PE could not be ruled out. Per study protocol, all suspected recurrent VTE events were evaluated by a central independent adjudication committee, including a radiologist, by directly comparing new diagnostic imaging against baseline films. To confirm a true new recurrence and distinguish it from previous thrombosis, the central independent adjudication committee required at least one of the following objective criteria: (1) a new intraluminal filling defect on CT pulmonary angiography (for PE) or an abnormal compression ultrasound in a previously normal vein segment (for DVT); or (2) at the index anatomical site, a substantial increase (≥4 mm) in thrombus diameter during full compression ultrasound, or a documented new extension or complete occlusion on follow-up imaging.

Major bleeding was defined according to the criteria of the International Society on Thrombosis and Haemostasis as overt bleeding associated with a fall in hemoglobin of ≥ 2 g/dL, or leading to a transfusion of ≥2 units of packed red blood cells or whole blood, or occurring in a critical site: intracranial, intraspinal, intraocular, pericardial, intra-articular, intramuscular with compartment syndrome, retroperitoneal, or contributing to death [16]. CRNMB was defined as overt bleeding not meeting the criteria for major bleeding but associated with medical intervention, unscheduled contact with a physician, (temporary) cessation of study treatment, or any other discomfort such as pain or impairment of activities of daily life [17].

### 2.1 Statistical analysis

At inclusion in the study, the index VTE was classified as either symptomatic or incidentally discovered. Fourteen of 298 patients were not registered for symptomatic or incidental VTE; thus, data from 284 patients were available for analysis. Baseline characteristics for registered patients are presented as frequencies (%) for categorical data and median for continuous data. Percentages are calculated on the total number of patients in each group. We compared the baseline characteristics of the patients by using the chi-squared test for categorical variables and the Mann–Whitney U-test for continuous variables. Major bleedings and CRNMBs were merged into 1 category, “Clinically relevant bleedings,” in some analysis, counting only 1 event per unique patient. Two patients, one in each VTE group, experienced both a CRNMB and a major bleed. For these individuals, the more severe event (major bleed) was prioritized to avoid double-counting. Time-to-event analyses were performed to evaluate clinical outcomes between the incidental and symptomatic VTE groups over the 36-month follow-up period. Death from any cause was treated as a key competing risk. Subdistribution hazard ratios (sHRs) with 95% CIs for VTE recurrence, clinically relevant bleeding, and their respective subgroups were calculated using the Fine-Gray competing risks model. All-cause mortality was compared between the groups using a standard Cox proportional hazards regression model to estimate the hazard ratio (HR). Risk factors for clinically relevant bleeding were evaluated using both univariable and multivariable Fine-Gray models. The key covariates included were high age, low hemoglobin, incidental VTE, low body mass index (BMI), presence of metastatic disease, concomitant ulcerogenic or bleed-inducing medications, and luminal gastrointestinal (GI) cancer, evaluated both as total luminal GI cases and specifically as unresected luminal GI cancer vs all other cancers. The final model included covariates in which the crude sHRs showed an association with bleeding (*P* < .05 or *P* ≈ .05). Low hemoglobin and low BMI were defined as values below the 20th percentile values in the nonbleeding patients. High age was defined as age above the 80th percentile in nonbleeding patients. Ulcerogenic or bleed-inducing medications included 5-fluorouracil, systemic corticosteroids, non-steroidal anti-inflammatory drugs, antiplatelet agents, and fluconazole. Luminal GI cancer included colorectal, small bowel, gastric, and esophageal cancer. Covariates were selected based on baseline imbalances or previously documented clinical associations with bleeding [[18], [19], [20], [21]]. The cumulative incidence over time for recurrent VTE and clinically relevant bleeding was estimated separately using cumulative incidence functions, with all-cause mortality treated as a competing risk. Differences between the cumulative incidence curves were evaluated using the Fine-Gray model. Statistical analyses were performed using Stata SE (version 17; StataCorp).

## 3 Results

Of 284 included patients, 163 (57.4%) had incidental VTE. Baseline characteristics of patients are shown in Table 1. Patients with incidental VTE had a lower BMI (24.3 vs 25.9, *P* = .002) and more often had metastatic disease (79.1% vs 57.0%, *P* < .001). Abdominal vein thrombosis (9.8% vs 0.8%, *P* = .002) and PE (73.0% vs 46.3%, *P* < .001) were more common in patients with incidental VTE, while DVT of the leg was more frequent among patients with symptomatic VTE (45.4% vs 7.4%, *P* < .001).

**Table 1:** Patient characteristics for symptomatic and incidental VTE (0-36 months).

Characteristics	Symptomatic VTE at inclusion (*n* = 121)	Incidental VTE at inclusion (*n* = 163)	*P* value
	*n* (%)/median (range)	*n* (%)/median (range)	
Age	66 (19-91)	69 (22-86)	.10
Gender, female	53 (43.8)	70 (42.9)	.88
BMI	25.9 (14.5-38.5)	24.3 (15.1-45.0)	.002
Metastatic disease	69 (57.0)	129 (79.1)	<.001
Previous VTE	12 (9.9)	14 (8.6)	.70
Index VTE			
Abdominal	1 (0.8)	16 (9.8)	.002
Cerebral veins	1 (0.8)	1 (0.6)	0.83
CVC	2 (1.6)	1 (0.6)	.40
Deep vein thrombosis arm	5 (4.1)	2 (1.2)	.12
Deep vein thrombosis leg	55 (45.4)	12 (7.4)	<.001
Pulmonary embolism	56 (46.3)	119 (73.0)	<.001
Other	1 (0.8)	12 (7.4)	.01
Primary cancer			
Colorectal	23 (19.0)	52 (31.9)	.01
Small bowel	2 (1.7)	5 (3.1)	.06
Upper gastrointestinal	2 (1.7)	10 (6.1)	.06
Pancreatic	7 (5.8)	18 (11.0)	.12
Cholangiocarcinoma	2 (1.7)	1 (0.6)	.40
Urogenital	23 (19.0)	32 (19.6)	.90
Hematological	17 (14.1)	9 (5.5)	.01
Breast	18 (14.9)	9 (5.5)	.01
Lung	10 (8.3)	16 (9.8)	.65
Melanoma	6 (5.0)	5 (3.1)	.41
Female genital	3 (2.5)	2 (1.2)	.43
Brain	4 (3.3)	0	.02
Other	4 (3.3)	4 (2.5)	.67

BMI, body mass index; CVC, central venous catheter; VTE, venous thromboembolism.

Patients with incidental VTE more often had colorectal cancer than patients with symptomatic VTE (31.9% vs 19.0%, *P* = .015). In contrast, patients with symptomatic VTE more often had hematological (14.1% vs 5.5%, *P* = .01) or breast cancers (14.9% vs 5.5%, *P* = .01).

Outcomes in patients with incidental and symptomatic VTE are presented in Table 2. Recurrent VTE during 36 months of anticoagulation therapy occurred in 9.8% of patients with incidental VTE and 6.6% of patients with symptomatic VTE (sHR, 1.5; 95% CI, 0.6-3.5). Clinically relevant bleeding was more common in patients with incidental VTE (24.5% vs 13.2%; sHR, 1.9; 95% CI, 1.1-3.4). All-cause mortality during the 36-month follow-up was 32.5% vs 28.9% in patients with incidental VTE and patients with symptomatic VTE, respectively (HR, 1.1; 95% CI, 0.7-1.7).

**Table 2:** Univariate analysis of bleeding and recurrent venous thrombosis in patients with symptomatic and incidental venous thromboembolism (0-36 months).

Outcomes	Symptomatic VTE at inclusion (*n* = 121), *n* (%)	Incidental VTE at inclusion (*n* = 163), *n* (%)	sHR (95% CI) in patients with incidental VTE compared with symptomatic VTE
Primary efficacy outcomes			
Total recurrent VTE	8 (6.6)	16 (9.8)	1.5 (0.6-3.5)
Symptomatic recurrence	5 (4.1)	6 (3.7)	1.3 (0.4-4.5)
Incidental recurrence	2 (1.6)	8 (4.9)	1.7 (0.4-6.5)
Unknown	1 (0.8)	2 (1.2)	
Types of recurrent VTE			
Pulmonary embolism	5 (4.1)	4 (2.4)	0.6 (0.2-2.2)
Symptomatic recurrence	3 (2.5)	1 (0.6)	
Incidental recurrence	1 (0.8)	3 (1.8)	
DVT lower extremity	1 (0.8)	3 (1.8)	2.3 (0.2-21.6)
Symptomatic recurrence	1 (0.8)	3 (1.8)	
Incidental recurrence	–	–	
DVT upper extremity	–	–	
Abdominal veins	1 (0.8)	6 (3.7)	4.5 (0.5-36.5)
Symptomatic recurrence	1 (0.8)	2 (1.2)	
Incidental recurrence	–	3 (1.8)	
Other	1 (0.8)	3 (1.8)	
Symptomatic recurrence	–	–	
Incidental recurrence	1 (0.8)	2 (1.2)	
Safety outcomes			
Clinically relevant bleeding^a^	16 (13.2)	40 (24.5)	1.9 (1.1-3.4)
Major bleeding	4 (3.3)	17 (10.4)	3.2 (1.1-9.6)
CRNMB	13 (10.7)	24 (14.7)	1.4 (0.7-2.8)
All-cause mortality	35 (28.9)	53 (32.5)	1.1 (0.7-1.7)
Cumulative outcomes^b^	22 (18.2)	54 (33.1)	1.9 (1.2-3.1)

CRNMB, clinically relevant nonmajor bleeding; DVT, deep venous thrombosis; sHR, subdistribution hazard ratio; VTE, venous thromboembolism.

a The number of patients with clinically relevant bleeding does not equal the mathematical sum of patients with major bleeding and CRNMB because 2 patients, 1 in each VTE group, experienced both types of events. For these individuals, only the major bleeding event was counted under clinically relevant bleeding to avoid double-counting.

b Included recurrent VTE and/or clinically relevant bleeding.

The time courses of the recurrent VTE and clinically relevant bleeding events for both full-dose and low-dose apixaban are shown in Figure. The incidental VTE group showed a slightly higher, but statistically nonsignificant, cumulative risk of recurrent VTE compared with the symptomatic VTE group during the 36-month intervention period (*P* = .34). The cumulative incidence of clinically relevant bleeding was higher in the incidental VTE group compared with the symptomatic VTE group (*P* = .027). A sharp increase in clinically relevant bleeding was observed in both groups during the initial conventional-dose phase (first 6 months). However, the risk remained consistently higher in the incidental VTE group throughout the study period.

**Figure:**
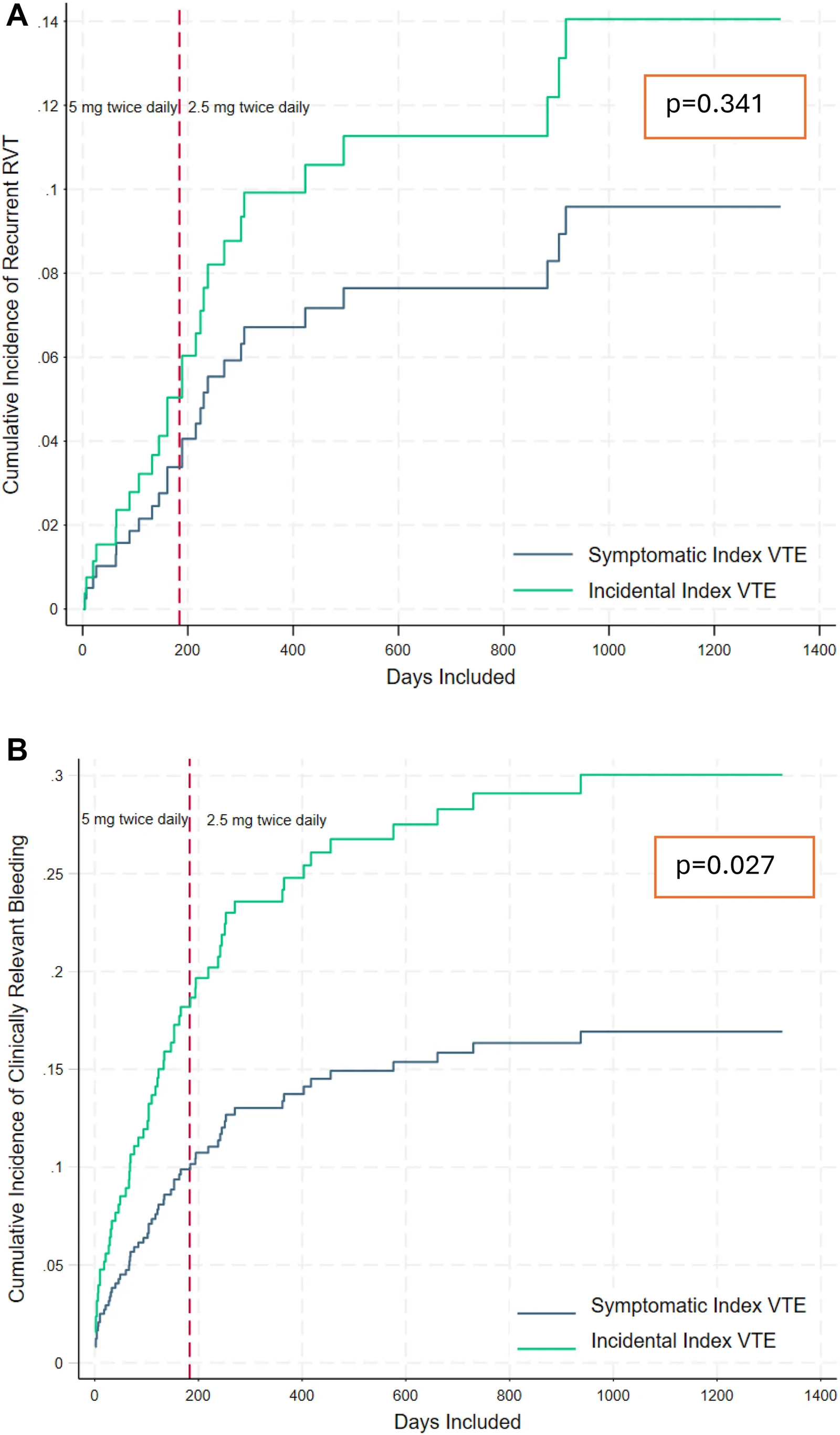
Recurrent VTE (A) and clinically relevant bleeding (B) in symptomatic vs incidental VTE in patients with cancer. VTE, venous thromboembolism.

In an effort to identify factors that could explain the association between incidental VTE and clinically relevant bleeding, we investigated several factors including GI cancer, low BMI, and low hemoglobin. We then included factors that were associated with bleeding in a multivariable model, but the association between clinically relevant bleeding and incidental VTE remained unchanged (sHR, 1.9; 95% CI, 1.1-3.4) (Table 3). We also tried to adjust for metastatic disease, GI cancer, and concomitant ulcerogenic or bleed-inducing medications, but it did not change this association.

**Table 3:** Multivariable Fine-Gray competing risks analysis for factors associated with clinically relevant bleeding (all-cause mortality as competing risk, 0-36 months).

Variable	Crude		Adjusted	
	sHR (95% CI)	*P* value	sHR (95%CI)	*P* value
Incidental VTE	1.9 (1.1-3.4)	.026	1.9 (1.1-3.4)	.030
Age >74 y (p80)	1.6 (0.95-2.8)	.077	1.7 (0.9-3.2)	.074
Hemoglobin below 10.5 (for females) and 11.1 (for males) (p20)	2.2 (1.3-3.7)	.002	2.5 (1.4-4.4)	.002
BMI below 21.7 (p20)^a^	1.9 (1.02-3.5)	.044	1.5 (0.8-2.9)	.238
Metastatic disease	1.2 (0.7-2.3)	.506	–	–
Concomitant ulcerogenic or bleed-inducing medications	0.8 (0.5-1.4)	.447	–	–
Luminal GI cancer vs all other cancers	0.8 (0.5-1.4)	.466	–	–
Unresected luminal GI cancer vs all other cancers	1.3 (0.7-2.5)	.406	–	–

p20: 20th percentile; p80: 80th percentile.

BMI, body mass index; GI, gastrointestinal; sHR, subdistribution hazard ratio; VTE, venous thromboembolism.

a Of 284 patients, 14 patients had missing data for BMI.

## 4 Discussion

In this study of cancer-related VTE, we found that clinically relevant bleeding was more common in patients with incidental VTE, whereas the recurrent VTE rate was comparable between the two groups. We also observed that the patients with incidental VTE were more likely to have lower BMI, metastatic disease, and abdominal VTE or PE as the index event, as well as a higher prevalence of colorectal and small bowel cancers. In contrast, patients with symptomatic VTE more frequently presented with DVT of the leg and had a higher prevalence of hematological or breast cancers.

Patients with incidental VTE exhibited a higher risk of bleeding, particularly during the initial high-dose anticoagulation treatment phase. This finding aligns with previous studies [9,10,22,23]. It has been speculated that the increased risk of bleeding observed in patients with incidental VTE might be attributed to the high proportion of colorectal cancer cases, as this cancer type is well known to be associated with a higher risk of bleeding during anticoagulation therapy [18,[24], [25], [26]]. However, our data did not support this hypothesis, as adjustment for GI cancer in the multivariable model did not attenuate the bleeding risk. Furthermore, to address potential confounding, we explicitly adjusted for high age, low hemoglobin, metastatic disease, low BMI, and concomitant ulcerogenic or bleed-inducing medications. Even after controlling for these factors, the bleeding risk observed in the incidental cohort remained increased and statistically significant. In our model, low hemoglobin levels and incidental VTE were independent risk factors for bleeding.

Our study found a slightly higher, though insignificant, rate of recurrent VTE in patients with cancer with incidental VTE. This is in contrast to previous reports, including a meta-analysis by Caiano et al [23], as well as reports by Mulder et al [9] and the Caravaggio study, which report numerically lower recurrence rates in incidental VTE [10]. This discrepancy may stem from differences in study populations, anticoagulation strategies, or cancer types. Notably, incidental VTE may involve nonthrombotic emboli (eg, tumor, septic, or fat emboli) or subacute/chronic thrombi, which can potentially reduce the recurrence risk [23,27].

The current results highlight different clinical features between patients with cancer with incidental and symptomatic VTE. The findings align with the Caravaggio study, which also reported differences in clinical characteristics between these two groups [10]. Hence, it is reasonable to view patients with cancer with symptomatic VTE and incidentally detected VTE as two different populations of patients. Similar to what was found in the Caravaggio study, we found that patients with incidental VTE often had a higher burden of metastatic disease, which is likely due to more frequent routine imaging for cancer staging in advanced disease, increasing the detection of asymptomatic thrombotic events. In addition, metastatic cancers are known to be more prothrombotic, which may further contribute to the increased incidence of VTE in patients with cancer [[28], [29], [30]].

It has been shown that many patients classified as having incidental VTE are not truly asymptomatic. The EPIPHANY study showed that patients with unsuspected PE who had symptoms despite being classified as incidental were more likely to have metastatic disease and higher mortality compared with patients with truly asymptomatic unsuspected PE and symptomatic PE [31]. O’Connell et al [27] also reported higher mortality among patients with symptomatic, unsuspected VTE compared with those with truly asymptomatic events. These findings support the notion that many cases classified as incidental VTE are not truly asymptomatic, as symptoms may often be overlooked or masked by cancer or its treatment. Thus, it is of some importance to distinguish between symptomatic and truly asymptomatic incidental VTE.

Cancer types differed between the two groups. Colorectal and small bowel cancers were more prevalent in incidental VTE, while hematological and breast cancers were more common in symptomatic VTE. This pattern is consistent with the findings in the Caravaggio study, which linked incidental VTE to colorectal cancer and symptomatic VTE to hematological cancers [10]. The anatomical proximity of colorectal tumors to major abdominal vessels may increase the likelihood of incidental VTE being visualized, particularly in the abdominal region. In addition, the frequent use of imaging for staging and follow-up in GI cancers, particularly colorectal cancer, increases the chances of detecting asymptomatic thrombotic events. The higher prevalence of symptomatic VTE in patients with hematological cancers may be related to the use of central venous catheters. In addition, incidental VTE may be underdiagnosed in hematological and breast cancers due to less frequent imaging.

A strength of our study is that, unlike retrospective studies on incidental VTE in patients with cancer, which are prone to bias, the CAP study’s prospective design improves the reliability of its conclusions. The study population in the CAP study more accurately reflects a real-world clinical setting than, for example, the prospective randomized Caravaggio study, which excluded patients with brain cancer and acute leukemia, included only DVT and PE cases, and involved only 20% incidental VTE. The CAP study included all VTE types with a nearly equal distribution of incidental and symptomatic cases [14,32]. The extended follow-up period (up to 36 months) further strengthens long-term outcome assessments, including recurrent VTE and bleeding complications.

While our findings align with previous studies, such as Caravaggio, enhancing their validity, the sample size may be insufficient to detect small yet clinically relevant differences. In addition, variability in diagnosing incidental VTE remains a challenge; despite using a standardized definition, subtle symptoms may blur the distinction between incidental and symptomatic VTE, potentially introducing bias or including clinically irrelevant cases. Furthermore, explicit data on participants’ race and ethnicity were not collected; however, the study population was predominantly of European descent, which reflects our regional clinical demographics but may limit generalizability to racially diverse cohorts. Lastly, although the study included a diverse cancer population, certain subgroups (eg, severe thrombocytopenia or specific cancer types) were under-represented, limiting generalizability to some high-risk populations.

## 5 Conclusion

Our study shows that VTE location and cancer type differ in incidental and symptomatically detected VTE. We also confirmed that incidental VTE is an independent risk factor for bleeding when treated with apixaban.
